# Interactive Effects of Filamentous Fungi and Cucurbitacin Phytonematicide on Growth of Cowpea and Suppression of *Meloidogyne enterolobii*

**DOI:** 10.3389/fmicb.2022.765051

**Published:** 2022-02-08

**Authors:** Kgabo Martha Pofu, Phatu William Mashela

**Affiliations:** Plant Production, Soil Science and Agricultural Engineering, Green Biotechnologies Research Centre of Excellence, University of Limpopo, Sovenga, South Africa

**Keywords:** arbuscular mycorrhizae, Biocult, cucurbitacin phytonematicides, *Glomus* species, *Meloidogyne enterolobii*, *Trichoderma* species

## Abstract

Cowpea [(*Vigna unguiculata* (L.) Walp)] is highly susceptible to the emerging guava root-knot nematode, *Meloidogyne enterolobii*, with available management options being limited due to the withdrawal of effective fumigant nematicides from the agrochemical markets. Filamentous fungi, available as Biocult (a.i. *Glomus* species + *Trichoderma asperellum* Lieckf and Nirenberg) and Nemafric-BL phytonematicide (a.i. cucurbitacin B) each improves plant growth and suppresses nematode population densities. However, when filamentous fungi like Biocult are combined with other biocontrol agents, the combined effects either have synergestic or antagonistic effects on the test variables. The combined effects of Biocult and cucurbitacin phytonematicides on plant growth and nematode suppression remain undocumented. The objective of this study was therefore to determine the combined effects of Biocult and Nemafric-BL phytonematicide on growth of cowpea var. Eureka and suppression of *M. enterolobii* population densities. Eureka was subjected to the effects of the two products in a 2 × 2 factorial experiment on a field infested with *M. enterolobii*. At harvest, the interaction of Biocult and Nemafric-BL phytonematicide was highly significant (*P* ≤ 0.01) on plant and nematode variables, with a two-way table used to assess the findings. Relative to untreated control, Biocult alone increased plant growth variables from 15 to 74%. Similarly, NemafricBL phytonematicide increased plant variables from 14 to 61%, whereas the combined effects significantly increased dry shoot mass (19%) and dry harvestable leaf mass (21%), but did not have significant effects on plant height and stem diameter. Relative to untreated control, Biocult alone reduced nematode eggs in root (80%), J2 in root (84%) and J2 in soil (53%), whereas the combined relative effects of the two products did not have significant effects on nematode population densities. In conclusion, Biocult and Nemafric-BL phytonematicide had antagonistic effects on growth of cowpea and suppression of *M. enterolobii* population densities and therefore, should be used separately in cowpea production.

## Introduction

In semi-arid regions, cowpea [(*Vigna unguiculata* (L). Walp] is one of the mandated crops for ameliorating food insecurity, where it is primarily cultivated for use as a leafy vegetable and secondarily as grains for consumption, feed and seed. Even though the crop is drought-tolerant, when cultivated as a low input crop, the productivity of the crop is substantially low due to an assortment of challenges, which include the availability of nutrient elements such as phosphorus and the incidence of high population densities of root-knot (*Meloidogyne* species) nematodes. Generally, *Meloidogyne* species are a serious threat in crop production systems, with crops without nematode resistance to the genus suffering losses from as high as 50% to total crop failure (Thies et al., 2016). Fortunately, certain cowpea cultivars/landraces with resistance to *Meloidogyne* species had been identified (Ononuju and Nzenwa, 2011; Ruanpanun and Somta, 2015). Nevertheless, the crop is also susceptible to cowpea aphid (*Aphis craccivora* Koch). Findings suggested that when infestations of phloem-sucking insects such as aphids and greenhouse whiteflies (*Trialeurodes vaporariorum* Westwood) when unchecked on sweet stem sorghum and wild *Cucumis* species, resistance to *Meloidogyne* species was invariably lost (Pofu et al., 2012; Mashela et al., 2017a; Maleka et al., 2021). Incidentally, another emerging *Meloidogyne* species, the guava root-knot nematode [*Meloidogyne enterolobii* (Jang and Eisenback)], with a wide range of host plants and, the shortest ontogeny of 15 days among thermophilic *Meloidogyne* species (Collet, 2020), was not affected by Mi resistance genes that affect most *Meloidogyne* species in *Solanum* species (Philbrick et al., 2020). Globally, *M. enterolobii* is being viewed as the most aggressive among *Meloidogyne* species (Castagnone-Sereno, 2012; Dareus et al., 2021). Due to the high damage impact induced by the genus, it is imperative that management strategies be in place for suppressing the population densities of this nematode to below the economic threshold level for crops without nematode resistance (Proveda et al., 2020).

Traditionally, nematode population densities were effectively managed using fumigant nematicides. However, their withdrawal from the agrochemical markets due to environmental issues that included climate change, shifted nematode management strategies to alternatives perceived as being sustainable and environment-friendly (Mashela et al., 2017b).

Filamentous fungi such as *Glomus* species (AMF) and *Trichoderma* species have capabilities for improving plant growth and suppressing various soil-borne pathogens, including plant nematodes (Baltruschat and Schonbeck, 1975; Richardson et al., 2011). Colonization of roots by *Glomus* species improve nutrient absorption, particularly phosphorus and certain micronutrient elements, along with conferring drought tolerance and disease resistance to plants (Ozbay and Newman, 2004; Chandanie et al., 2009). In contrast, colonization of roots by *Trichoderma* species confer resistance to pathogens, including plant nematodes (Vasundhara et al., 2016; Proveda et al., 2020). In root-free soil, *Trichoderma* species had antagonistic effects on AMF (Green et al., 1999). On tomato plants, *T. feltiae* and cucurbitacin phytonematicides had synergistic effects on plant growth variables and suppression of *M. incognita* population densities under greenhouse conditions (Madaure et al., 2019). As noted in other reports, the efficacy of *Trichoderma* species is both fungus-specific and plant-specific, with numerous contradicting effects (Stewart and Hill, 2014; Proveda et al., 2020).

The cucurbitacin phytonematicides, Nemarioc-AL and Nemafric-BL phytonematicides, with active ingredients cucurbitacin A (C_32_H_46_O_9_) and cucurbitacin B (C_32_H_46_O_8_), respectively, are being developed from fruits of indigenous wild *Cucumis* species to South Africa as alternatives to fumigant nematicides (Mashela et al., 2017a). The two phytonematicides each consistently improved plant growth and suppressed nematode population densities of various *Meloidogyne* species under a wide range of conditions (Mashela et al., 2017a). Efficacies of the two phytonematicides were comparable to those of the carbamate and organophosphate nematostatic products (Mashela et al., 2008). Filamentous fungi and cucurbitacin phytonematicides are ideal for use as cost-effective products in low-input crops such as cowpeas. Biocult WS (Biocult Pty, Sommerset West, South Africa) is a South African product, with each gram containing 400 propagules of *Glomus* species + 1 × 10^9^ CFU *Trichoderma asperellum*. Lieckf and Nirenberg. Although both Biocult and cucurbitacin phytonematicides are separately being researched and developed for improving plant growth and suppressing nematode population densities. Generally, the combined effects of filamentous fungi with other agricultural biocontrol agents have had antagonistic effects (Proveda et al., 2020). The combined effects of the two test variables on leguminous crops and suppression of nematodes had not been documented. The objective of this study was therefore to investigate the interactive effects of Biocult and Nemafric-BL phytonematicide on growth of cowpea and suppression of *M. enterolobii* population densities under field conditions.

## Materials and Methods

### Study Location

The trial was conducted in autumn (March-May) 2019 and repeated in 2020 under field conditions at the Green Biotechnologies Research Centre of Excellence, University of Limpopo (23°53′10″S, 29°44′15″E), South Africa. The location is semi-arid with summer rainfall averaging less than 450 mm per annum and summer temperature averaging 28^°^C. Hutton soil at the experimental site comprised 65% sand, 30% clay, 5% silt, which was thus classified as loam, with pH (H_2_O) 6.5 and EC 1.87 dS/m.

### Treatments and Experimental Design

Nemafric-BL phytonematicide was prepared through fermentation using oven-dried fruit from cultivated wild watermelon (*Cucumis africanus* L.) and effective microorganisms (EM) as described previously (Mashela et al., 2017a). Biocult and Nemafric-BL phytonematicide, as the first and the second main factors, respectively, were laid out in a 2 × 2 factorial experiment. The four treatments (B_0_P_0_, B_1_P_0_, B_0_P_1_, B_1_P_1_) were arranged in randomized complete block design with 10 replications (*n* = 40), with blocking being for shading by windbreaks in the morning and in the afternoon. Due to the factorial nature of the experiment, there was no need to repeat the experiment (Little and Hills, 1978).

### Cultural Practices

A drip irrigation line was laid out resulting in planting stations at 1.0 m × 0.45 m spacing, with each drip hole discharging 2 L chlorine-free water/h. A day before sowing, each station was irrigated with 3 L half-strength Hoagland solution without N (Hoagland and Arnon, 1950). Primed cowpea var. Eureka seeds were inoculated with *Rhizobium leguminosarium biovar phaseoli* spores while still moist and placed on laboratory bench for 3 days prior to sowing. Four seeds were sown per drip hole and at 5 days after complete emergence, seedlings were thinned to one per drip hole.

### Initiation of Treatments

Soil samples were collected adjacent to each seedling using a Soil Sampler Probe 52 cm (Mylawncare, Johannesburg, South Africa), composited and mixed. Second-stage juveniles (J2) were extracted from 250 ml soil subsample using the modified sugar-centrifugation floatation method (Marais et al., 2017), with J2 counted under a 60 × magnification stereomicroscope, with mounted specimen identified as *M. enterolobii* using morphometric characters. The initial nematode population density (Pi) averaged 35 J2/250 ml soil subsample, which necessitated augmentation of each seedling with 250 eggs + J2 *M. enterolobii* collected from roots of tomato plant cv. “Floradade.” Eggs + J2 were dispensed using a 20 ml plastic pipette around each seedling with holes covered using soil. Five days after thinning, Biocult was applied by placing 2 g in a shallow furrow around each seedling and covering with soil. Five days thereafter, Nemafric-BL phytonematicide was applied at 2%, with biweekly reapplication until harvest. Aphid populations were monitored daily, with plants sprayed once at 35 days after seedling emergence using malasol at 5 ml/5 L chlorine-free water to contain the aphid infestations.

### Data Collection and Analysis

At 60 days after the phytonematicide treatment, whole plants were removed. Plant length was measured from the severed end to the tip of the flag leaf of the longest vine using a measuring tape. Stem diameter was measured at 5 cm above the severed distal end using a digital Vernier caliper. Tender leaves usually harvested for use as leafy vegetable were harvested and dried with shoots in air-forced ovens at 60°C for 72 h for dry leaf and shoot mass. Root systems were removed from each station, immersed in water to remove soil particles, blotted dry, with eggs and J2 extracted from 10 g roots using the modified sugar floatation method (Marais et al., 2017). From each planting site, 300 ml soil sample was collected, mixed and J2 extracted from a 250 ml soil subsample (Marais et al., 2017), with root and soil nematodes separately counted under a stereomicroscope. Nematode data were transformed using log_10_(x + 1) to normalize the variances. All datasets were separately subjected to the Shapiro-Wilk test using Statistix 10 software to assess the normality of the distribution of data (Shapiro and Wilk, 1965; Ghasemi and Zahedias, 2012). The data exhibited normal distribution and were therefore subjected to factorial analysis of variance.

## Results

The seasonal interactions for variables were not significant and data were pooled (*n* = 80) and reanalyzed. The interactions on plant and nematode variables were highly significant (*P* ≤ 0.01), and therefore the data were expressed using a two-way table (Little, 1981), with treatment means compared at the probability level of 5% using Tukey HSD All-Pairwise Comparison test. Due to the factorial nature of the study, there was no need to validate the results Little and Hills, 1978). Unless otherwise stated, only treatment effects which were significant at 5% level of probability were discussed.

### Plant Growth Variables

At harvest, the interaction of Biocult and Nemafric-BL phytonematicide was highly significant (*P* ≤ 0.01) on plant growth variables. Relative to untreated control, Biocult alone increased plant variables from 15 to 74% (Table 1). Similarly, NemafricBL phytonematicide increased plant variables from 14 to 61%. However, the combined effects significantly increased dry shoot mass by 19% and dry harvestable leaf mass by 21%, but did not have significant effects on plant height and stem diameter.

**TABLE 1:** Relative impact (RI) of Biocult (B) and Nemafric-BL phytonematicide (P) on dry harvestable leaf mass (DHLM), dry shoot mass (DSM), plant height (PHT), and stem diameter (STD) of cowpea var. Eureka at 56 days after the treatments under field conditions (*n* = 80).

Biocult																
DSM (kg)^y^					DHLM (kg)				PHT (cm)				STD (mm)			
BL	B_0_	RI (%)^z^	B_1_	RI (%)	B_0_	RI (%)	B_1_	RI (%)	B_0_	RI (%)	B_1_	RI (%)	B_0_	RI (%)	B_1_	RI (%)
P_0_	3.71^c^	–	6.45^a^	74	0.97^b^	–	1.40^a^	44	25.05^b^	–	28.81^a^	15	7.51	–	8.14	11
P_1_	5.98^a^	61	4.43^b^	19	1.32^a^	37	1.17^a^b	21	28.61^a^	^14a^	25.81^a^	3	8.30	14	7.53	–0.4
Standard error = 1.07					Standard error = 0.96				Standard error = 1.12				Standard error = 1.89			

*^y^Means with the same letter within the variable were not different (P ≤ 0.05) according to Tukey test.*

*^z^RI (%) = [(Treatment/Control) – 1] × 100.*

### Nematode Suppression

The interaction of the two products was also highly significant on nematode variables Relative to untreated control, Biocult alone reduced nematode eggs in root, J2 in root and J2 in soil by 80, 84, and 53%, respectively (Table 2). However, the combined effects of the two products did not have significant effects on any of the test nematode variables in roots, but significantly reduced J2 in soil by 30%.

**TABLE 2:** Relative impact (RI) of Biocult (B) and Nemafric-BL phytonematicide (P) on eggs in roots, second-stage juveniles (J2) in root, J2 in soil on cowpea var. Eureka at 60 days after the treatments under field conditions (*n* = 80).

Biocult												
	Eggs/10 g root^y^				J2/10 g root				J2/250 ml soil			
Nemafric-BL	B_0_	RI (%)^z^	B_1_	RI (%)	B_0_	RI (%)	B_1_	RI (%)	B_0_	_*RI* (%)_	B_1_	RI (%)
P_0_	954^a^	–	188^c^	–80	3,490^a^	–	560^b^	–84	2,094^a^	–	977^b^	–53
P_1_	103^b^	–89	594^a^	–38	210^c^	–94	27 95^a^	–20	90	–96	1,459^c^	–30
Standard error = 2.11				Standard error = 1.86					Standard error = 3.12			

*^y^Means with the same letter within the variable were not different (P < 0.05) according to Tukey test.*

*^z^RI (%) = [(Treatment/Control) – 1] × 100.*

## Discussion

### Plant Growth

In the current study Biocult and Nemafric-BL phytonematicide each significantly increased growth variables of cowpea plants. *Glomus* species and *Trichoderma* species in Biocult have the potential of increasing soil biological activities that improve soil health by inducing the root system to exudate various secondary metabolites, including enzymes, into the rhizosphere (Stirling, 2014; Yan et al., 2021). Additionally, *Glomus* species, the arbuscular mycorrhizal fungi, provide benefits that include improved absorption of phosphorus and certain micronutrient elements (Richardson et al., 2011) and nematode suppression (Gough et al., 2020). The species can also accord the host plant the ability to access water beyond the normal wilting point, thereby according the plants the drought tolerance status, particularly under suboptimal conditions (Proveda et al., 2020).

Another endophytic fungi, *Trichoderma* species, in addition to promoting plant growth through various mechanisms (Ozbay and Newman, 2004), which include mineral solubilization and boost nutrient uptake, also provide certain degree of protection against soil pathogens, including nematodes (Stewart and Hill, 2014). Colonization of roots by *Trichoderma* species had been associated with improved chlorophyll content, alleviation of abiotic stress, increased exudation of secondary metabolites and the production of various plant growth regulators hormones (Yan et al., 2021). However, the effects of *Glomus* species and *Trichoderma* species on plant growth are plant- and species-specific (Stewart and Hill, 2014; Gough et al., 2020). Combined, *Glomus* species and *T. asperellum* in Biocult were previously observed to have synergistic effects on increasing dry shoot mass in cucumber (*Cucumis sativa* L.) plants when compared with individual treatment effects (Chandanie et al., 2009).

In our study, Nemafric-BL phytonematicide had consistent effects on improving growth variables of cowpea plants. In other studies, where the product was used to manage nematode population densities of *M. incognita* and *M. javanica*, the product consistently improved crop growth variables (Mashela et al., 2017a). Cucurbitacins in phytonematicides can either have stimulation, neutral or inhibition effects on plant growth. However, since the dosage accumulates in the rhizosphere with repeated phytonematicide application, response in different plant organs can also be concentration-specific as shown in different studies (Mashela et al., 2017a). Cucurbitacin B in Nemafric-BL phytonematicide is able to penetrate the apoplastic pathways in roots, but cannot penetrate the symplastic pathway which is accorded by the pericycle and the endodermis in root systems (Van Wyk and Wink, 2014). In our study, as shown by the plant growth responses, concentration of cucurbitacin B in apoplastic spaces remained within the stimulation range for all the test plant variables since growth was significantly increased.

Notably, the combined effects of Biocult and Nemafric-BL phytonematicide, had antagonistic effects on plant growth when compared with that of either of product. In colonized root systems, mycelia of the filamentous fungi penetrate the root system to establish mutual relationships (Stewart and Hill, 2014; Proveda et al., 2020). Such relationships change the morphology and metabolism of the root system, along with the bioactive chemical compounds of affected plant roots (Vasundhara et al., 2016; Yan et al., 2021). Apparently, when the two products are combined, there are both physical and chemical limitations in roots. Firstly, the proliferation of mycelia in the apoplast spaces, alter the available physical spaces in which free water that carries cucurbitacin molecules perculate. Consequently, the reduced free-passage of free water, invariably results in reduced concentration of cucurbitacin B in apoplastic spaces. Secondly, although reduced cucurbitacin B could have stimulation effects on plant growth variables, for lower organisms such as *Glomus* and *Trichoderma* species, the concentration could have been at the inhibition range, technically, the cytotoxic range. Under *in vitro* conditions, which mimic apoplastic conditions, *T. harzianum* suppressed mycelia of *G. intraradices* (Green et al., 1999).

### Nematode Suppression

Cowpea var. Eureka is a host to *Meloidogyne* species (Mashela and Pofu, 2012). In our study, Biocult and Nemafric-BL phytonematicide each consistently suppressed nematode population densities of *M. enterolobii* in root and in soil, which confirmed observations in other studies (Mashela et al., 2017a; Proveda et al., 2020; Yan et al., 2021). *Trichoderma* species are primarily biocontrol agents that have inherent capabilities of triggering plants to release chemicals with nematicidal properties (Proveda et al., 2020; Yan et al., 2021). Such chemicals include flavonoids, phenols and chitinase, β-1,3 glucanase, protease, amylase, which are known as plant genes that confer nematode resistance in plants (Mashela et al., 2017b). The induced plant resistance as observed in var. Eureka in our study, had been referred to as systemic acquired resistance (Stirling, 2014; Proveda et al., 2020). The effects of Nemafric-BL phytonematicide on population densities of *M. enterolobii* in the current study, confirmed those of this product on a wide range of other *Meloidogyne* species and races (Mashela et al., 2017b), which we do not wish to discuss further in the current study.

Both *Glomus* species and *Trichoderma* species are endophytes, with different roles and capabilities of inducing nematode resistance in plants (Proveda et al., 2020). However, the two species, when combined, were at times shown to have significant antagonistic effects on population densities of *Meloidogyne* species (Yan et al., 2021), with the effects not being limited to the induced chemicals. After J2 hatch, J2 move out of roots into soil solution, from where search for penetration sites occurs. After penetration, J2 move to the tip of root and enter the vascular bundle and then move upward through the xylem to the zone of differentiation, where feeding sites are established (Mashela et al., 2017b). In addition to inducing chemicals with nematicidal properties (Yan et al., 2021), mycelia and spores of the endophytes can physically impede J2 mobility (Stewart and Hill, 2014; Gough et al., 2020; Proveda et al., 2020).

Generally, the effects of cucurbitacins on entities are both concentration- and size-specific. Concentrations that have stimulation effects on plant growth were previously shown to have significant inhibition effects on various bioactivities on minute entities like plant nematodes (Dube, 2016). In Nemarioc-AL phytonematicide, with active ingredient cucurbitacin A, which is partly polar and therefore slightly soluble in water. Nodules induced by *Bradyrhizobium japonicum* on cowpea vs. increasing concentration of Nemarioc-AL phytonematicide exhibited quadratic positive relationships, with nodules being highly sensitive to the product (Kola et al., 2018). Although in the current study we observed consistent antagonistic effects of Biocult and Nemafric-BL phytonematicide, *T. harzianum* in short-term studies was tolerant to Nemarioc-AL and Nemafric-BL phytonematicides at concentrations used in nematode management (Madaure et al., 2019). In our study, Nemafric-BL phytonematicide was applied biweekly, with the likelihood that the stable cucurbitacin B accumulated within the apoplastic spaces, thereby inhibiting both mycelia and spore development. In other studies (Vasundhara et al., 2016; Proveda et al., 2020) the combined effects of *Trichoderma* species with either *Paecilomyces lilacinus* biocontrol agent, neem (*Azadirachta indica* A. Juss) phytonematicide or castor (*Ricinus communis* L.) oil had synergistic effects in suppressing population densities of *Meloidogyne* species, which contradicted observations in our study. However, our findings confirm the assertion that the efficacy of filamentous fungi on inducing beneficial effects is dependent upon the fungal species, plant species and pathogen type and species (Gough et al., 2020). Additionally, in interaction with agricultural remedies of botanical nature, the degree of sensitivity of filamentous fungi to the active ingredients of the test product could also play an indispensable role in the expression of the interactive effects.

## Conclusion

Biocult and Nemafric-BL phytonematicide each consistently promoted growth of cowpea var. Eureka and suppressed population densities of *M. enterolobii* in both root and soil. In contrast, the combined effects of the two products had antagonistic effects on growth of the test plant and suppression of nematode population densities. In conclusion, the two products should be applied separately in cowpea production until the issues of antagonism are resolved.

## Data Availability Statement

The original contributions presented in the study are included in the article/supplementary material, further inquiries can be directed to the corresponding author/s.

## Author Contributions

KP conducted the field experiments over two seasons and analyzed the data. Both authors contributed equally to the discussion of the results, formulated the objectives, and designed the study.

## Conflict of Interest

The authors declare that the research was conducted in the absence of any commercial or financial relationships that could be construed as a potential conflict of interest.

## Publisher’s Note

All claims expressed in this article are solely those of the authors and do not necessarily represent those of their affiliated organizations, or those of the publisher, the editors and the reviewers. Any product that may be evaluated in this article, or claim that may be made by its manufacturer, is not guaranteed or endorsed by the publisher.
